# Application of biostimulant products and biological control agents in sustainable viticulture: A review

**DOI:** 10.3389/fpls.2022.932311

**Published:** 2022-10-18

**Authors:** Keiji Jindo, Travis L. Goron, Paloma Pizarro-Tobías, Miguel Ángel Sánchez-Monedero, Yuki Audette, Ayodeji O. Deolu-Ajayi, Adrie van der Werf, Misghina Goitom Teklu, Moshe Shenker, Cláudia Pombo Sudré, Jader Galba Busato, Raúl Ochoa-Hueso, Marco Nocentini, Johan Rippen, Ricardo Aroca, Socorro Mesa, María J. Delgado, Germán Tortosa

**Affiliations:** ^1^ Agrosystems Research, Wageningen University and Research, Wageningen, Netherlands; ^2^ Department of Plant Agriculture, University of Guelph, Guelph, ON, Canada; ^3^ Faculty of Computer Sciences, Multimedia and Telecommunication, Universitat Oberta de Catalunya (UOC), Barcelona, Spain; ^4^ Department of Soil and Water Conservation and Organic Waste Management, Centro de Edafología y Biología Aplicada del Segura (CEBAS), Agencia Estatal CSIC, Murcia, Spain; ^5^ School of Environmental Sciences, University of Guelph, Guelph, ON, Canada; ^6^ Chitose Laboratory Corp., Kawasaki, Japan; ^7^ The Robert H. Smith Faculty of Agriculture, Food and Environment, Rehovot, Israel; ^8^ Laboratório de Melhoramento Genético Vegetal, Universidade Estadual do Norte Fluminense Darcy Ribeiro, UENF, Campos dos Goytacazes, Brazil; ^9^ Faculdade de Agronomia e Medicina Veterinária, Campus Universitário Darcy Ribeiro, Universidade de Brasília, Brasília, DF, Brazil; ^10^ Department of Biology, IVAGRO, Agroalimentario, Campus del Rio San Pedro, University of Cádiz, Cádiz, Spain; ^11^ Department of Terrestrial Ecology, Netherlands Institute of Ecology (NIOO-KNAW), Wageningen, Netherlands; ^12^ Dipartimento di Scienze e Tecnologie Agrarie, Alimentari, Ambientali e Forestali (DAGRI), Università degli Studi Firenze, Firenze, Italy; ^13^ Wijngaard El Placer, Lelystad, Netherlands; ^14^ Department of Soil Microbiology and Symbiotic Systems, Estación Experimental del Zaidín (EEZ), Agencia Estatal CSIC, Granada, Spain

**Keywords:** biological agents, biostimulants, grapevine, organic, vineyard, viticulture, wine

## Abstract

Current and continuing climate change in the Anthropocene epoch requires sustainable agricultural practices. Additionally, due to changing consumer preferences, organic approaches to cultivation are gaining popularity. The global market for organic grapes, grape products, and wine is growing. Biostimulant and biocontrol products are often applied in organic vineyards and can reduce the synthetic fertilizer, pesticide, and fungicide requirements of a vineyard. Plant growth promotion following application is also observed under a variety of challenging conditions associated with global warming. This paper reviews different groups of biostimulants and their effects on viticulture, including microorganisms, protein hydrolysates, humic acids, pyrogenic materials, and seaweed extracts. Of special interest are biostimulants with utility in protecting plants against the effects of climate change, including drought and heat stress. While many beneficial effects have been reported following the application of these materials, most studies lack a mechanistic explanation, and important parameters are often undefined (e.g., soil characteristics and nutrient availability). We recommend an increased study of the underlying mechanisms of these products to enable the selection of proper biostimulants, application methods, and dosage in viticulture. A detailed understanding of processes dictating beneficial effects in vineyards following application may allow for biostimulants with increased efficacy, uptake, and sustainability.

## Introduction

Grape (*Vitis vinifera*) production has increased globally over the last decade from 65 million to 77.8 million tons. The world’s vineyards totaled 7.4 million hectares in 2018, of which ~4.5% were cultivated with organic approaches (i.e., refraining from pesticides and chemical fertilizer use and promoting sustainable cultivation practices). About 88% of organic vineyards are located in Europe, primarily in France, Italy, and Spain. There is a need, and increasing demand, for more ecologically-sustainable agricultural products as outlined by the United Nations Sustainable Development Goals (SDGs). For production to support demand despite current anthropogenic climate change (Roka, 2022), more research on sustainable food systems is urgently needed.

Higher yields are necessary for sustainable food security on a finite land basis (Godfray et al., 2010; Foley et al., 2011). According to Renaud-Gentié (Seufert et al., 2012), the yield ratio between organic and conventional agricultural production per unit area is 0.75. To close this yield gap while simultaneously satisfying consumer preference for organic products, improved techniques for organic grape farming are required.

Additionally, grape varieties often require specific environmental conditions, historically located in very narrow latitudinal zones (30–50°N and 30–40°S) with minimal temperature extremes (Nesbitt et al., 2018). Due to the high environmental sensitivity of *Vitis vinifera*, a strong correlation between meteorological conditions propelled by global warming (e.g., heat waves, extreme precipitation, droughts, hailstorms, and windstorms) and low grape yield and wine quality has been reported (Santos et al., 2020).

Biostimulants are substances or microorganisms applied to plants, which enhance nutrition use efficiency, abiotic stress tolerance, and crop quality traits, using mechanisms that do not directly add fertility to the soil (du Jardin, 2015), and in the EU the marketing of biostimulants is regulated by specific provisions (Regulation EU 1009, 2019). Initially, these technologies were primarily utilized in organic farming, with later uptake in both conventional and integrated systems (Rouphael and Colla, 2020). The application of biostimulant technologies is now a well-known tool, though poorly-researched strategy to enhance plant growth and prevent pests and diseases (Gutiérrez-Gamboa et al., 2019; Cataldo et al., 2022; Monteiro et al., 2022; Olavarrieta et al., 2022).

Biocontrol agents (BCAs) are organisms antagonistic to crop pests. Synthetic pesticides, mainly fungicides, are widely used in viticulture. However, pesticide residue contamination of grapes and grape products (raisins, juices, and wines) is widely studied and may be detrimental to consumer health (Walorczyk et al., 2011). An EU report (EFSA, 2021) has shown increased pesticide residue levels in primary commodity food products since 2016. Table grape products are of particular concern, in which 66% of products were contaminated, and 14.3% of products tested contained more than five different pesticide residues. In a sample of dried vine fruits, 28 pesticides were detected (Cabrera and Pastor, 2021). When testing grape leaves, 37.9% had residues above the allowed maximum residue levels (MRL). Gava et al. (2021) highlighted the importance of tracing contamination from the originating vineyard to the wine. Of additional concern is that these residues can affect alcoholic fermentation, change product flavor, and pose a toxicological risk to the consumer as well as those involved in the production chain.

**Figure 1:**
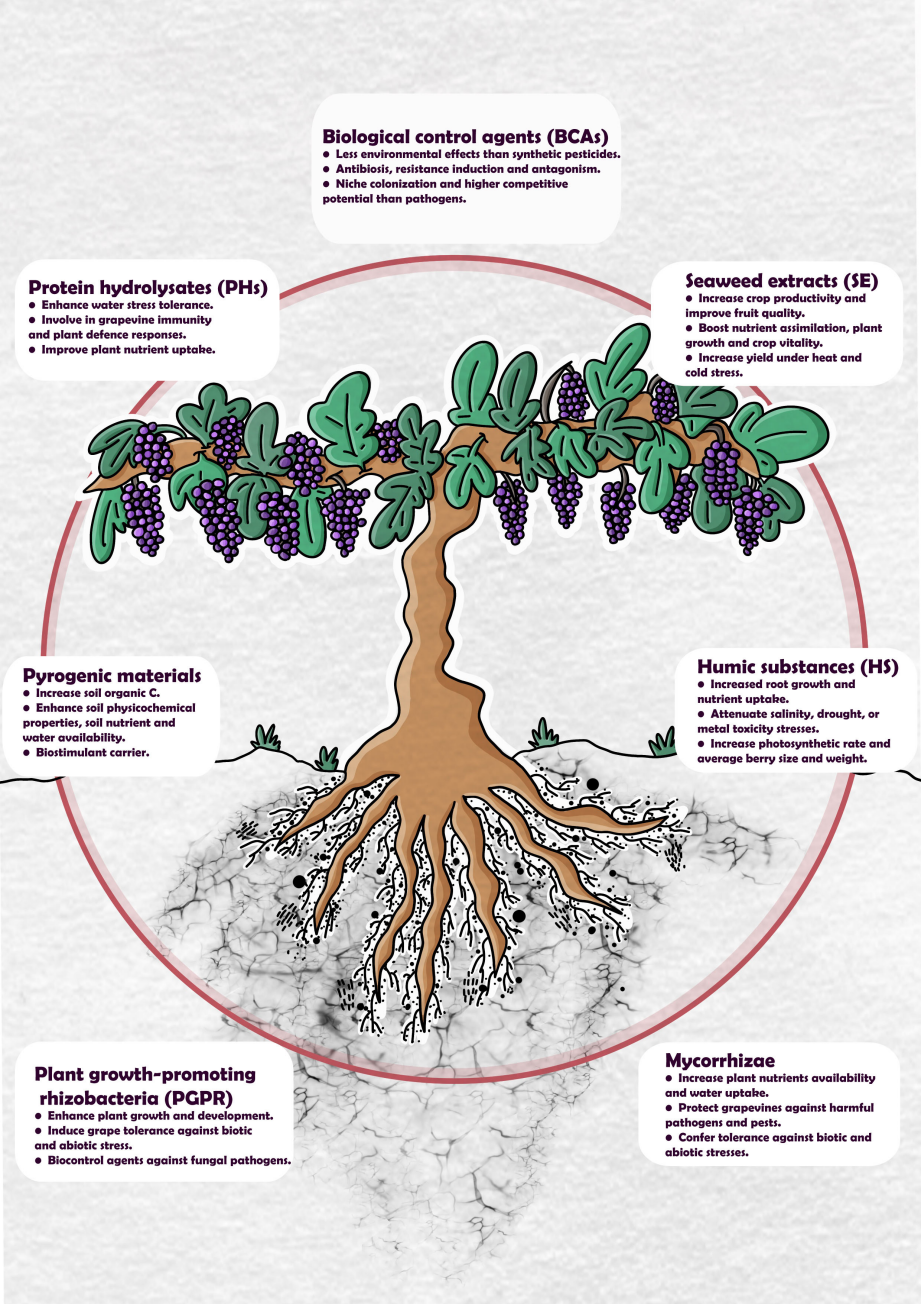
An overview of the main effects of biological control agents (BCAs), protein hydrolysates (PHs), seaweed extracts (SE), pyrogenic materials, humic substances (HS), plant growth-promoting rhizobacteria (PGPR), and mycorrhizae applied in viticulture.

This review presents a series of different biostimulant materials and biological control agents utilized in sustainable vineyards (their main effects on *Vitis vinifera* are summarized in **Figure 1**). Additionally, the origin of the materials, the application methods, and the underpinning functions/mechanisms of each material are described. The limitations of current practices and alternative approaches to aid future studies of the interaction between viticulture and global climate change are also addressed.

## Biostimulant materials

### Protein hydrolysates

Protein hydrolysates (PHs) refer to mixtures of polypeptides, oligopeptides, and amino acids that are manufactured from protein sources using partial hydrolysis (Schaafsma, 2009). They are produced by either acid and/or alkaline hydrolysis (Schaafsma, 2009), enzymatic hydrolysis (Colla et al., 2015), or from by-products of plant or animal origins (Meggio et al., 2020). In horticulture, more than 90% of PH utilized is derived from animal origin, while plant-derived PHs are relatively novel (Colla et al., 2015). Animal-derived PHs often contain higher amounts of total amino acids compared to plant-derived PHs (Ertani et al., 2009). Plant-derived PHs contain carbohydrates and phenols, which enhance plant oxidative stress defenses, as well as aid in energy metabolism (Colla et al., 2015). PHs can be further subdivided into two categories: (1) protein fractions with a relatively high content of specific amino acids, such as glutamine peptides and cysteine/glycine and tryptophan peptides; and (2) bioactive peptides with specific amino-acid sequences (i.e., 3 to 20 amino acids), which are usually hydrophobic (Schaafsma, 2009). In general, the effects of PHs on plant physiology are closely related to the stimulation of carbon and nitrogen metabolism, hormone activity, and nutrient supply and uptake by plants (**Figure 1**). Moreover, it has been demonstrated that PHs can stimulate the plant microbiome which can promote plant growth and development by enhancing water and nutrient uptake as well as their adaptation to biotic and abiotic stresses (Colla et al., 2017, and sources reviewed within).

The effects of PHs on grapevine growth are reviewed in **Table 1**. PHs are known to regulate gene expression involved in the transport of nutrients, and the signaling and metabolism of reactive oxygen species, thereby enhancing plant stress tolerance (Meggio et al., 2020). For example, PHs can effectively improve grapevine tolerance to water deficit (Boselli et al., 2019; Meggio et al., 2020). A study by Boselli et al. (2019) showed that PH-treated grapevines contain higher levels of anthocyanins, often observed under water stress conditions (Castellarin et al., 2007). Therefore, PHs may be able to mimic water stress conditions in grapevine and reduce water loss due to evapotranspiration (Boselli et al., 2019).

**Table 1:** An overview of the main effects of protein hydrolysates (PHs) applied in viticulture.

Crop variety	Applied material and properties	Dosage, the form of application, and stage of plant development	Experimental condition	Effects	Reference
Table grape inoculated with either B. cinerea (100 conidia/wound) or natural infections.	- PH derived from soybean. - PH derived from casein.	- 20 µL in the wound (3 x 3 mm) 24 h before inoculation. - Spraying at preharvest (0.8 and 1.6 g L^-1^ on vines every 15 d) or a combination of a pre- (0.8 g L^-1^) and postharvest treatment (200 mL).	-Organic vineyard cultivation during the 2012/2013 season, planted in 1998 in Fumane, Valpolicella, Verona, Italy.	- Significant reduction of gray mold incidence by 67% (soybean PH) and 54% (casein PH) at a low application dose (0.8 g L^-1^). - In the field, PH derived from either soybean or casein reduced gray mold incidence by 65 and 92% respectively compared to water control.	Lachhab et al. (2016).
Grapevine plantlets (*Vitis. Vinifera cv* Marselan) inoculated with *Plasmopara. viticola.*	- PH derived from soybean. - PH derived from casein.	- 10 mL of each PH per plant was sprayed on 8-week-old grapevine plantlets.	-Greenhouse conditions.	- Application of PH derived from either soybean or casein reduced downy mildew infected leaf surface by 76% and 63% as compared to the control, respectively. - Both PHs acted as elicitors to enhance grapevine immunity against pathogen attack.	Lachhab et al. (2014).
Grape cultivar *(Vitis Vinifera L., cv* Corvina).	- PH derived from soybean. - PH derived from casein. - PH derived from lupin.	-Spraying at two concentrations; 1.6 and 6.4 g L^-1^ at every 10 d three times, from fruit-set to bunch closure.	- Field experiments were carried out over 5 growing seasons (2012 to 2016) in an organically managed vineyard of *Vitis vinifera L.* cv. Corvina, 24-year-old vines located in the Valpolicella area, Italy.	- All treatments significantly ameliorated the total anthocyanin content of berries compared to the control. - The greatest effect was obtained by soybean-derived PH. - All PHs reduced plant water loss.	Boselli et al. (2019).
Grapevine *(Vitis Vinifera L., cv Sauvignon Blanc).*	- A novel PH biostimulant (APR).	-A concentration of 0.5 g L^-1^ PH was added to the soil or pot as a soil drench.	-Pot experiment approximating field-conditions in a tunnel at the “L. Toniolo” Experimental Farm of the University of Padua in Legnaro, NE Italy in 2018.	- Application of PHs to roots before imposing water deprivation mitigated consequences of stress by sustaining the growth of younger vegetative organs, and limiting the extent of cell dehydration.	Meggio et al. (2020).
Grapevine (*Vitis Vinifera* cv. Marselan) inoculated with *Plasmopara viticola.*	- Laminarin (Lam), a ß-1,3 glucan polymer from the algae *Laminaria digitata* - The chemically sulfated form of Lam, PS3.	-Lam and PS3 solution (5 g L^-1^) were sprayed on upper and lower leaf faces until the run-off point.	-Greenhouse pot experiment	- PS3 triggered a long-lasting plasma membrane depolarization of grapevine cells. - PS3 and Lam shared a common stress-responsive transcriptome profile, which resulted in induced resistance against *P. viticola.*	Gauthier et al. (2014).
Grapevine (*Vitis vinifera L.*).	- nutrient broth PH – a natural derivative from peptone *via* meat (1.9 g L^-1^) and yeast extract (0.7 g L^-1^).	- 5.0 (2010), 3.0 (2011), and 3.0 g L^-1^ (2013) of PH -Treatment was applied weekly with a motorized backpack mist blower.	-Field trials were carried out in 2010, 2011, and 2013 at S. Michele all’Adige (Italy) in a vineyard planted in 1997.	-PH controlled powdery mildew on grapevine across seasons (comparable to standard fungicide treatments) both on leaves and bunches across three different years.	Nesler et al. (2015).

In addition, PHs act as signal compounds to trigger plant defense responses (Boller, 1995). Grapevine is highly susceptible to various pathogens (Lachhab et al., 2014). In particular, downy mildew (*Plasmopara viticola*) and powdery mildew (*Erysiphe necator*) are the most widespread and devastating grapevine diseases worldwide (Gauthier et al., 2014; Nesler et al., 2015). Frequent fungicide applications (i.e., every 7-10 days in season) are often required to control these diseases, a practice which not only dramatically increases production costs but also negatively impacts the environment and human health (Jones et al., 2014). Plant defense by-products may be an attractive alternative to chemical pesticides (van Aubel et al., 2014). Elicitors including PHs are not toxic to plants and are recognized by plant membrane receptors, meaning they are often freely able to mobilize an array of plant defenses (Boller and Felix, 2009). PHs contain a large variety of bioactive peptides, which may act as plant growth regulators, antioxidants, and biostimulants (Colla et al., 2014), as well as directly influence numerous biological processes evoking hormonal and immunological responses (Ito et al., 2006; Phelan et al., 2009). Therefore, PHs can trigger signaling events involved in grapevine immunity (Gauthier et al., 2014). Some studies (Gauthier et al., 2014; Lachhab et al., 2014) report reduced symptoms of downy mildew and gray mold (*Botrytis cinerea*) on grapevine following applications of PHs derived from soybean and casein. PHs may improve plant nutrient uptake through modification of root architecture, by complexation of nutrients such as Zn (Ertani et al., 2018), or stimulation of microbial and enzymatic activities (Colla et al., 2015) – e.g., increased activity of N_2_-fixing, P-solubilizing, and indoleacetic acid-producing bacteria was observed in lettuce (Colla et al., 2015) and tomato (Colla et al., 2014) after application of PHs. The application of PHs also increases the activity of both nitrate reductase and glutamine synthetase in roots and leaves (Ertani et al., 2009). Nitrate reductase reduces nitrate to ammonia, and glutamine synthetase catalyzes the ATP-dependent amination of glutamate to produce glutamine, thereby facilitating assimilation or re-assimilation of nitrogenous sources originating from a wide variety of anabolic or catabolic processes (Ertani et al., 2009). Applications of different PHs have been observed to increase grapevine yield, correlated with PH organic N content (Bosselli et al., 2019).

Further research on the effects of PHs on viticulture should be explored, especially the impact on microbial communities living in the rhizosphere or phyllosphere (aboveground plant surfaces inhabited by microbes), which has received little study (Colla et al., 2015 and Colla et al., 2017).

### Seaweed extracts

Biostimulants can also be produced from marine macroalgae (i.e. seaweed extracts; SEs). In the last decade, the application of SEs in agriculture has gained traction and several seaweed species (such as brown seaweeds *Ascophyllum nosdosum*, *Ecklonia maxima*, and *Laminaria* spp, and red seaweeds *Kappaphycus alvarezii*, and *Gracilaria edulis*) are currently used in commercial production of biostimulants (Yakhin et al., 2017; El Boukhari et al., 2020). SEs have been successfully explored in arable cropping, including viticulture (**Table 2**). The most common method of applying SEs in vineyards is foliar spraying, although the extracts can be applied directly to the soil (Mancuso et al., 2006; de Carvalho et al., 2019). Like most biostimulants, SEs may increase crop productivity and improve fruit quality in both stressed and non-stressed conditions (Deolu-Ajayi et al., 2022). The vegetative shoot growth stage of the crop is frequently targeted for SE application to stimulate growth and boost crop vitality by modulating underlying biochemical processes, which are also important at the reproductive stage (**Figure 1**). SEs may also enhance microbial biodiversity, but information about this effect on the plant microbiome is scarce (El Boukhari et al., 2020).

**Table 2:** An overview of seaweed-based (SEs) biostimulants applied in viticulture.

Crop variety	Product details and seaweed species	Bioactive substance	Experimental conditions	Application and dosage	Physiological effects	References
*Vitis vinifera* L. cv. Karaerik	Commercial biostimulant “Maxicorp” containing *Ascophyllum nodosum*.	Not Specified.	Greenhouse experiment with 1-year-old grapevine saplings grown in 20 cm diameter pots. Four increasing fertilization levels, including a control without any nutritional element, were used.	Foliar spray of seaweed biostimulants at individual concentrations of 0.5, 1, and 2 g L^-1^. A total of 4 applications occurring every 15 days were performed. The initial treatment was applied to 2-week-old plants.	Increased macro- (nitrogen, phosphorus, potassium, calcium, and magnesium) and micronutrient (iron, zinc, magnesium, and copper) plant uptake.	Turan and Köse (2004).
	Commercial biostimulant “Proton” (seaweed species not specified).					
	Commercial biostimulant “Algipower” (seaweed species not specified).					
*Vitis vinifera* L. cv. Sangiovese.	Not specified.	IPA (Indole-3- propionic acid) extract.	Greenhouse experiment with 1-year-old *V. vinifera* cv. Sangiovese grafted onto *V. berlandieri* x *riparia* ‘420A’ in 25 cm^2^ pots. Water stress was introduced by stopping irrigation for 6 days and restoring it after the dry period.	Biostimulant was applied at a rate of 10 g L^-1^, with foliar sprays occurring 2 times weekly for the duration of the experiment. *A fertilizer NPK 9-5-4 was combined with the biostimulant treatment.	Significant increase in nitrogen, phosphorus, potassium, and magnesium uptake, and also in total dry weight. In water deficit conditions, plants showed faster recovery of their leaf water potential.	Mancuso et al. (2006).
*Vitis vinifera* L. cv. Niágara Rosada.	Commercial biostimulant “Acadian LSC” containing *A. nodosum*.	Not Specified	A field trial in an established vineyard in Lavras, Brazil. The vineyard was cultivated with the espalier method and has clayey soil.	Foliar application of the biostimulants at 6 g L^-1^. Each biostimulant was applied 4 times: 20 days after dormancy, at blooming, fruit set, and veraison.	Significant increase in potassium, boron, and zinc; as well as an estimated yield increase of 24.15% (equivalent to 2.72 t ha^-1^) compared to the control.	de Carvalho et al. (2019).
	*Hypnea musciformis.*				Significant increase in magnesium and zinc, but no significant increase in crop yield.	
	*Lithothamnium* sp.				Significant increase in boron only, and a 13.17% yield increase equivalent to 1.46 t ha^-1^.	
	*Sargassum vulgare*.				Significant increase in magnesium, but no significant increase in crop yield.	
*Vitis vinifera* L. cv. Sangiovese.	A commercial biostimulant “Acadian marine plant extract powder” containing *A. nodosum.*	Not specified.	Field trial (2013) in Umbria, Italy growing only the Sangiovese cultivar. The vineyard has a vertical shoot-positioned trellis cultivation system, and loamy soil.	- Foliar application for all 3 cultivars at 1500 g ha^-1^, 5 times during the growing season, beginning 2 weeks before grape veraison and occurring every 10-14 days. - An additional treatment with 3000 g/ha of the biostimulant was performed on only the Sangiovese cultivar.	Improved fruit color of the Sangiovese cultivar.	Frioni et al. (2018).
*Vitis vinifera* L. cv. Pinot Noir.			Field trial (2014) in an established vineyard with both Pinot Noir and Cabernet Franc, located in Benton Harbour, Michigan, USA. The vineyard has a vertical shoot-positioned trellis cultivation system, and sandy loam soil.		Increased vegetative parameters of both grape cultivars. Shortened ripening time in only Pinot Noir.	
*Vitis vinifera* L. cv. Cabernet Franc.						
*Vitis vinifera* L. cv. Red globe.	Application of commercial biostimulant “SUNRED” containing an unspecified seaweed extract.	Not specified.	Field trial (2015/2016) in Pengshan county, Meishan, China. The cultivation method and soil were not specified.	Foliar biostimulant treatments of 0.6, 0.8, and 10 g L^-1^ were each applied twice: at the start of veraison and 5 days later.	Improved grape color at the different biostimulant concentrations.	Deng et al. (2019).
*Vitis vinifera* L. cv. Chardonnay.	Commercial biostimulant “Seasol” containing brown seaweeds *Durvillaea potatorum* and *A. nodosum*.	Not specified.	Field trials (2012/2013 and 2013/2014) near Kenley, Australia. The vineyard uses a double-wire cordon trellis system of cultivation and has calcareous loamy sandy soil.	Drip irrigation of 10 L ha^-1^ of biostimulant per application, with 8 and 4 applications at the 1^st^ and 2^nd^ field trials respectively. Biostimulant was first applied at the woolly bud and budburst growth stages respectively, and treatments occurred every 20-30 days.	Significant increase in grape yield by 13.8% (equivalent to 1.6 t/ha) compared to the control.	Arioli et al. (2021).
*Vitis vinifera* L. cv. Semillon.			Field trials (2012/2013 and 2013/2014) near Balranald, Australia. The vineyard uses a double-wire cordon trellis system, and has calcareous loamy sandy soil.	Drip irrigation of 5 L ha^-1^ of the biostimulant with 3 applications in both field trials. Treatments were applied at the budburst, flowering, and fruit set growth stages.	The yield increased by 10% (equivalent to 1.3 t/ha) compared to the control.	
*Vitis vinifera* L. cv. Merlot.			Field trial (2013/2014) near Loxton, Australia. The vineyard uses a double-wire cordon trellis system and its soil type is calcareous loamy sand.	Drip irrigation of 5 L ha^-1^ of the biostimulant with 3 applications. Treatments were applied at the budburst, flowering and fruit set growth stages.	The yield increased by 17.6% (equivalent to 1.8 t ha^-1^) compared to the control.	
*Vitis vinifera* L. cv. Merlot.			Field trial (2014/2015) at Tharbogang, Australia. The vineyard uses a single-wire cordon trellis system and has duplex soils ranging from loamy sand to clay loam.	Drip irrigation of 10 L ha^-1^ of the biostimulant with 4 applications. Treatments were applied at the budburst, flowering, fruit set, and veraison growth stages.	The yield increased by 11% (equivalent to 0.5 t ha^-1^) compared to the control.	
*Vitis vinifera* L. cv. Cabernet Sauvignon.			Field trial in (2016/2017) at Tharbogang, Australia. The vineyard uses a double-wire cordon trellis system and has duplex soils ranging from loamy sand to clay loam.	Drip irrigation of 10 L ha^-1^ of the biostimulant with 3 applications. Treatments were applied at 4-cm growth, flowering, and fruit set.	The yield increased by 10.7% (equivalent to 0.7 t ha^-1^) compared to its control.	
*Vitis vinifera* L. cv. Montepulciano.	Not specified.	Laminarin.	Field trials (2012 and 2013) at Camerano, Italy grown according to the Guyot trellis system. Biostimulant treatment started at the inflorescence growth stage.	Foliar spraying with 1 L ha^-1^ of commercial biostimulant “Frontiere” in each of the 11 treatments, and treatments occurred weekly.	The biostimulant had no significant reduction of grapevine downy mildew.	Romanazzi et al. (2016).
*Vitis vinifera* L. cv. Moscato.	Not specified.	Laminarin.	Greenhouse conditions with ~ 60-day old transplanted grape plants in 4 L pots. Plants were inoculated twice with powdery mildew pathogen (*Erysiphe necator*).	- Treatment with 2 g L^-1^ of a commercial biostimulant “Vacciplant” per timepoint. Biostimulants were applied at 6 time points occurring every 7-9 days: 2 treatments occurred prior to 1^st^ pathogen inoculation, another 2 before 2^nd^ pathogen inoculation, and 2 last treatments after the 2^nd^ pathogen inoculation. - A fungicide methiram was simultaneously used on the plants.	Reduction in Powdery mildew disease severity in the grape plants.	Pugliese et al. (2018).
			Field trials (2016 and 2017) at Piedmont, Italy, in an established vineyard with an espalier cultivation method and “Guyot” pruning.	Treatment with 2000 g/ha per timepoint. Biostimulant treatment was applied at 9 individual time points, occurring every 8-10 days. - A fungicide methiram was simultaneously used on the plants.		

Physiologically, SEs boost nutrient assimilation and plant growth under non-stressed conditions. Spraying wine grapevines with any of three commercial SEs (Maxicrop, Proton, and Algipower) increased both macro- and micro-nutrient uptake from the soil (Turan and Köse, 2004; **Table 2**). Similar mineral uptake induction in wine grapevines was reported by Mancuso et al. (2006) and de Carvalho et al. (2019) when SEs were applied, resulting in a subsequent increase of dry weight by ~ 27% and fruit yield by ~ 24% (**Table 2**). Foliar application of either an *Ascophyllum*-derived seaweed extract or the commercially available extract “SUNRED” improved fruit quality and shortened the ripening time of wine grape (Frioni et al., 2018; **Table 2**) and table grape (Deng et al., 2019; **Table 2**) cultivars. The enhancement in fruit quality may be linked to an accumulation of anthocyanins and phenolic compounds, especially in the berry skin, which was also observed (Frioni et al., 2018; Deng et al., 2019).

During drought stress and water deprivation in controlled greenhouse experiments, foliar application of a seaweed indole-3-propionic acid (IPA)-derived extract induced faster recovery of leaf water potential and stomatal kinetics (Mancuso et al., 2006). Different wine grape cultivars treated with a commercial seaweed biostimulant “Seasol” *via* soil fertigation, showed an average fruit yield increase of 14.7% across five locations with varying environmental conditions (Arioli et al., 2021; **Table 2**). Yield increase occurred irrespective of heat and/or cold stress in a single growing season (Arioli et al., 2021). The performance of the field experiments suggests an essential soil-crop interaction for increased productivity (Arioli et al., 2021). Unfortunately, analyses of the soils and the applied extracts were not reported and therefore, cannot support this hypothesis. Moreover, variations in the SE dosage in different fields in this study prevent further conclusions regarding the direct effect soil type (and properties) may have on SE, as well as the overall impact on crop productivity in the presence of the biostimulant.

Although SEs reduce the effects of crop diseases (Shukla et al., 2019) and non-seaweed-derived biostimulants also contribute to disease prevention in viticulture (reviewed in Gutiérrez-Gamboa et al., 2019), the specific use of SEs to mitigate diseases of grapevine has not been extensively studied. Treatment with a commercial seaweed-derived biostimulant “Vacciplant” caused a reduction in the effects of powdery mildew disease in both greenhouse experiments and field trials (**Table 2**). Grapevine disease severity was reduced by ~ 65% and 52% in greenhouse and field experiments, respectively (Pugliese et al., 2018). Thus, there is still potential for the application of seaweed-derived biostimulants in viticulture for disease control, especially considering the need for reduced agrochemical reliance in the coming decades.

Bioactive compounds have biological activity in plants, some of which result in increased crop productivity and resilience to stress. While the presence of bioactive compounds in seaweed has long been suspected of having stimulatory effects on plants, including complex polysaccharides (absent in arable crops, e.g. alginate, laminarin, and fucoidan), phytohormones, sterols, and osmolytes (Yakhin et al., 2017), speculations remain regarding underlying mechanisms. This is perhaps due to current research focusing on growth promotion, rather than also investigating and linking the role of these bioactive compounds to specific mechanisms. Further research is needed, and will also provide essential information for biostimulant optimization. Of note is that changes in mineral concentration due to foliar application of SEs do not always enhance crop productivity. Only two of four SEs tested caused a significant increase in grapevine yield (de Carvalho et al., 2019; **Table 2**), indicating that SEs must be tested independently to establish their benefits. Conflicting observations may be explained by variations in biochemical composition among seaweed species and their derived extracts, linked to the environmental conditions of the cultivation sites as well as harvesting times (Khairy and El-Shafay, 2013). Overall, the application of SE is beneficial for crops in both stressed and non-stressed conditions and thus may be attractive for wider adoption in viticulture.

### Humic substances

Humic substances (HS) are an important carbon compartment present in soils, waters, and sediments. Although researchers are not in complete agreement as to how HS are formed and structured, a growing body of evidence indicates they may serve as biostimulants of plant growth. HS biostimulants are available commercially in the form of products based on humic acids (HA), fulvic acids (FA), or a mixture of both (HA+FA). Efficacy is related to the product’s chemical composition, the form of extraction and application, the applied concentration, and the stage of development of the crop (Canellas et al., 2009; Zandonadi et al., 2013; Olivares et al., 2017; Jindo et al., 2020). Effects of HS application in plants often include the activation of plasma membrane H^+^-ATPase and the alteration of the primary and secondary metabolism, generally resulting in increased root growth, nutrient uptake, rate of photosynthesis, and attenuation of stress associated with salinity, drought, or metal toxicity (Zandonadi et al., 2007; Dobbss et al., 2018; Jindo et al., 2020) (**Figure 1**). It is suggested that some biostimulant effects of HS originate from their impact on structural and physiological modifications in roots and shoots related to nutrient assimilation and soil distribution (Canellas et al., 2015). Also, HS can simultaneously regulate the transcription and activity of some plant hormones following addition to the rhizosphere (Souza et al., 2022). Recently, it has been demonstrated that HS can alter the plant microbiome by favoring the recruitment of beneficial microorganisms after application (da Silva et al., 2021).

Despite a limited number of studies involving the specific use of HS in grape cultivation, several effects are consistently observed (**Table 3**). These include increases in photosynthetic rate, total soluble solids, total chlorophyll, and average berry size and weight (Ferrara and Brunetti, 2008 and Ferrara and Brunetti, 2010; Ibrahim and Ali, 2016; Popescu and Popescu, 2018; Irani et al., 2021). For example, Popescu and Popescu (2018) noted increased photosynthetic rate and total soluble fruit solids in cultivars ‘Feteasca Regala’ and ‘Riesling Italian’ planted in Romania, following foliar application of HA (40 and 50 mL L^-1^) previously extracted from vermicompost. The increase was attributed to a higher concentration of carotenoids in the leaves. Ferrara and Brunetti (2010) reported an improvement of parameters associated with fruit quality (e.g. increase in total soluble solids, [°Brix], and the °Brix/titratable acidity ratio, and a decrease in tartaric acid) due to the application of HA (100 mg L^-1^, extracted from the soil in Apulia, Italy) at full-bloom. In the cultivar ‘Italy’, six applications of HA (derived from different origins) increased chlorophyll content, °Brix value, and berry size (Ferrara and Brunetti, 2008). The authors attributed these effects to the presence of 6% nitrogen in the humic matrix. However, the increase in berry size might also be the result of the hormone-like activity of HA (i.e., auxin, gibberellin- and cytokinin-like activity), which is useful for organic seedless table grape production where the application of synthetic hormones is not permitted. In Egypt, Ibrahim and Ali (2016) applied HA (Greenhum Company, Italy) in both February and March to the cultivar ‘Superior Seedless’. Chlorophyll content, berry weight, and total fruit volume significantly increased after HA was respectively applied to leaves (5.0 g L^-1^) or to the soil (5 and 7.5 g L^-1^, 2.5 g L^-1^). In Spain, Sánchez-Sánchez et al. (2006) reported increased iron and phosphorus levels and reduced sodium in the leaves of cultivar ‘Italy’ following treatment with solutions containing HA+FA. Recently in Iran, Irani et al. (2021) applied FA (0.5%) *via* leaf spray or HA (20 g plant^-1^) *via* irrigation, and observed the alleviation of drought stress, increased nutrient absorption, and concentration of proline (an important stress signaling molecule), carbohydrates, and soluble proteins. Additionally, HA and FA application resulted in greater berry weight, productivity, and concentration of total soluble solids (Irani et al., 2021).

**Table 3:** An overview of the main effects of humic substances (HS) applied in viticulture.

Crop variety	Applied material and properties	Dosage, the form of application, and stage of plant development	Experimental condition	Effects	Reference
*Vitis vinifera* cv. Italia.	HA+FA commercial (Mol). Functional groups (with Nuclear Magnetic Resonance ^13^C): 90.7% of fulvic acids with three main portions that consisted of 10% aliphatic C, 73% aromatic C and 16.9% carboxylic C.	Two applications were carried out on the soil, in mid and late March.	Field cultivation. Twelve-year-old plants; drip-irrigated.	An increase in Fe and P absorption, as well as a decrease in Na absorption; Increased berry weight.	Sánchez-Sánchez et al. (2006).
*Vitis vinifera* cv. Italia. Grapevines were grafted onto 1103 P (*V. berlandieri* x *V. rupestris*).	HA was extracted from soil or organic compost. 54.91% of C; 4.91% of H; 6.73% of N; 32.67% of O; C/N ratio of 9,52; Total acidity of 5.0 meq g^-1^ of C; Phenolic acidity of 2.2 meq g^-1^ of C and carboxyl 2.7 meq g^-1^ of C	5 and 20 mg L^-1^, *via* leaf spray. Six applications with a 21-day interval.	Field cultivation. Forty-five-year-old plants; drip-irrigated.	Increased berry size and reduction in titrable acidity; Increase in N content, total chlorophyll content, yield, and soluble solids (°Brix).	Ferrara and Brunetti (2008).
*Vitis vinifera* cv. Feteasca Regala (henceforth RF) and cv. Riesling Italian (henceforth RI). Grapevines were grafted on rootstock hybrid Kober 5 BB (*Vitis berlandieri* x *Vitis riparia*).	Commercial HA (BioHumusSol Company Ltd.). 14.5 g humic substances L^-1^, 19 ppm nitrate nitrogen (NO_3_-N), 104 ppm ammonium nitrate (NH_4_-N), 22.5 ppm P, 132 ppm K, 39 ppm Ca, 75 ppm Mg, 75 ppm Na	30, 40, and 50 mL L^-1^, *via* leaf spray, one application at the pre-bloom and fruit set the phenological stage.	Field cultivation. Non-irrigated.	Increase in the total leaf area, yield, and total soluble solids (50 mL L^-1^); Increase in photosynthesis rate, chlorophyll a and b, and carotenoids (40 e 50 mL L^-1^)	Popescu and Popescu (2018).
*Vitis vinifera* cv. Italia. Grapevines were grafted onto 1103 P (*V. berlandieri* x *V. rupestris*).	HA extracted from soil. 52.46% of C; 5.77% of H; 5.4% of N; 35.66% of O; C/N ratio of 11.33; Total acidity of 5.0 meq/g of C; Phenolic acidity of 2.2 meq/g of C and carboxyl 2.7 meq/g of C	100 mg L^-1^ *via* spray foliar.	Field cultivation. Drip-irrigated.	Increased berry weight; Increase in total soluble solids (°Brix); decrease of tartaric acid.	Ferrara and Brunetti (2010).
*Vitis vinifera* cv. Superior seedless.	Commercial HA (Humatic 8500).	One, two, or three applications.	Field cultivation. Eleven-year-old plants; drip-irrigated.	Increase in total soluble solids, yield, and fruit quality with four HA applications.	Ibrahim and Ali (2016).
*Vitis vinifera* cv. Yaghouti’.	HA (K_2_O 10%; HA 55%; FA 15%) or FA Total nitrogen 3%; K_2_O 10%; FA 50%; chlorine 4.3%.	Application of HA with irrigation water at a concentration of 20 g per vine, two times (bud swell and millet- sized berry). Application of FA as a foliar spray at a concentration of 0.5% two times (millet-sized berry and 2 weeks later).	Field cultivation. Ten years old (plants); drip-irrigated.	Increase in berry weight, yield, total soluble solids, proline, and soluble carbohydrates (both HA and FA; under water stress conditions or not). Increase in N, P, K, Zn, and Fe content (leaf). In the plants with and without water stress, only the N content was not increased.	Irani et al. (2021).

Protocols outlining best practices in terms of vineyard HS application practices and optimal concentrations are yet to be developed, and research regarding potential negative structure-activity interactions has yet to be conducted. Despite these shortcomings, the beneficial effects of HS on grapevines are evident, generally resulting in increased production, increased fruit quality, and an expansion of the plant’s ability to tolerate environmental stresses.

### Pyrogenic materials

Pyrogenic materials are obtained by pyrolysis of plant biomass, a thermal decomposition process occurring at relatively high temperatures in the absence of oxygen. These materials represent a sustainable source of biostimulants with a long tradition in agriculture (Ogawa and Okimori, 2010). Pyrogenic materials include biochar (solid C-rich residue of pyrolysis), and pyroligneous acid (also known as wood vinegar, the aqueous fraction obtained from the condensation of pyrolysis vapors). Biochar is highly aromatic, porous, and possesses a chemically recalcitrant structure. It is generally used as a soil amendment, due to potential agronomic and environmental benefits associated with enhanced soil fertility and long-term soil C sequestration (Woolf et al., 2010; Lehmann et al., 2011). Pyroligneous acid consists of a mixture of a large number of oxygenated organic compounds (including acids, alcohols, ketones, phenols, furans, and ethers) and hydrocarbons with antioxidant and antimicrobial properties. Pyroligneous acid has potential as a plant growth and germination biostimulant, antioxidant and free-radical scavenger, pesticide, and antimicrobial agent (Mungkunkamchao et al., 2013; Grewal et al., 2018).

Biochar is more commonly used than pyroligneous acid in vineyards, either alone or in combination with compost (**Figure 1** and **Table 4**). There is contrasting information in the literature regarding biochar’s impact on vineyard productivity. A recent meta-analysis by Payen et al. (2021) showed that biochar application increased soil organic C, at a rate of 8.96 Mg CO_2_-eq ha^−1^ yr^−1^. The authors also postulated biochar application might lead to enhanced vineyard productivity, but additional long-term investigations are needed to support this statement. Several authors (Baronti et al., 2014; Genesio et al., 2015; Maienza et al., 2017) have reported increased productivity in biochar-amended vineyards, associated with enhanced physicochemical properties of the amended soils, increased soil water availability, and growth of fine root biomass (Amendola et al., 2017). However, there were no significant differences in grape quality. Conversely, other reports indicate only minor effects on soil physicochemical properties following biochar application over a three-year period, which did not significantly affect vineyard fertility (Schmidt et al., 2014; Sánchez-Monedero et al., 2019). Although these authors did observe benefits following the addition of biochar-blended compost to the soil, effects were similar to those in control vineyards amended with conventional compost without biochar.

**Table 4:** An overview of the main effects of pyrogenic materials in viticulture.

Crop variety	Pyrogenic material	Properties	Application and dosage	Experimental condition	Effects	Reference
*Vitis vinifera* L. (cv. Pinot Noir).	Biochar from 80% various hardwoods and 20% various coniferous wood chips at 750°C.	Soil organic amendment.	8 t ha^−1^ of biochar (d.w.).63.2 t ha^−1^ biochar-compost (d.w.), biochar added at 20% before composting process.	Field trial (3 years),rainfed vineyard (25-35 years old).	No relevant effects on plant growth parameters of vine or vine health are caused by biochar and biochar-compost.Minor effects on grape quality only in the first year of trial.	Schmidt et al. (2014).
*Vitis vinifera* L. (cv. Merlot, clone 181; rootstock 3309 Couderc).	Biochar from orchard pruning at 500°C.	Soil organic amendment.	16.5 t ha^−1^ of biochar (d.w.) applied in two consecutive years.	Field trial,rainfed vineyard (20 years old).	After 3 years: Increased soil water holding capacity and plant available water content.	Baronti et al. (2014).
					After 5 years: Increased vineyard production with no detrimental effects on grape quality.	Genesio et al. (2015).
					After 5 years: No negative impact on soil microbial community, and no retention of toxic compounds (PAH and heavy metals).	Maienza et al. (2017)
					After 7 years: Biochar is effective in restoring degraded soil functionality. The effects persist after 7 years of following a one-time application.	Giagnoni et al. (2019).
*Vitis vinifera* L. (cv. Grenache).	Biochar from grapevine trunks at 550°C.	Soil organic amendment.	5 t C ha^−1^ of biochar.5 t C ha^−1^ biochar-compost.	Field trial (2 years),rainfed vineyard (20 years old).	Improvement in the N nutrient status of vines after application of ‘compost’ and ‘compost x biochar’ treatments in year two.	Ubalde et al. (2014).
*Vitis vinifera* L. (cv. Pinot Blanc).*Vitis vinifera* L. (cv. Ribolla Gialla).*Vitis vinifera* L. (cv. Sauvignon).	Biochar from oak at 650°C.	Soil organic amendment.	10.9 t C ha^−1^ of biochar (d.w.).10.9 t C ha^−1^ of biochar: compost (d.w.), biochar added at 10% before composting process.	Field trial (3 years),3 rainfed vineyards.	Biochar enhanced soil water availability, but had no significant effects on soil nutrient availability, grape yield, or must quality.Biochar-blended compost had a similar effect as conventional compost.	Sánchez-Monedero et al. (2019).
*Vitis vinifera* L. (cv. Shiraz)	Not available.	Soil organic amendment.	8.5 t ha^−1^ of biochar.	Field trial (4 years),rainfed vineyard	No significant effect on yield and vegetative growth	Botelho et al. (2020)
*Vitis vinífera* ssp. *vinífera.*	Biochar from maize corn cob rachis at 450 -500°C.	Soil organic amendment.	5 t C ha^−1^ of biochar.	Field trial (2 years),rainfed vineyard (25 years old).	Reduced soil microbial biomass for at least two years after application.No significant effects on the community composition of soil microbes or microarthropods.	Andrés et al. (2019).
*Vitis vinifera* L. (cv. Montepulciano).	Biochar from orchard pruning at 500°C.	Soil organic amendment.	10 t ha^−1^ of biochar (d.w.).	Field trial (1 year),rainfed vineyard (15 years old).	Increased organic carbon, available water content, and formation of a large fraction of macro aggregates.Increased fine root biomass with no significant effect on the production of arbuscular mycorrhizal fungi.	Amendola et al. (2017).
*Vitis vinifera* L. *(*cv. Chardonnay).	Biochar from orchard pruning at 500°C.	Soil organic amendment.	30 t ha^−1^ biochar (fresh weight).	Rhizobox.	Enhanced soil physicochemical soil properties and increased soil water content during the harsh summer period.Promoted earlier root production and lowered the number of fibrous roots.	Montagnoli et al. (2021).
*Vitis rotundifolia* L. (Muscadine grape cv. Alachua).	Biochar from southern yellow pine at 400°C.	Soil organic amendment.	0, 5%, 10%, 15%, and 20% of biochar (d.w.).	Greenhouse study in pots.	Enhanced soil water and nutrient status, and improved plant P and Mg uptake, with no significant differences in plant physiological performance.	Chang et al. (2021a).
					Improved soil physical properties and stimulation of fine root development.	Chang et al. (2021b).
*Vitis vinifera* L.	Biochar from dairy manure at 400°C.	Soil organic amendment.	2 g and 5 g of biochar kg^-1^ soil.	Pot experiment (2 months).	Enhanced plant growth and soil properties under water stress conditions.	Kanwal et al. (2018).
*Vitis vinifera* L. (cv. Pinot noir).	Not available.	Soil organic amendment.	2% of biochar (d.w.).	Pot experiment.	Enhanced soil water availability under water shortage.No significant impact on soil N availability.	Petrillo et al. (2020).
*Vitis vinifera* L.	Biochar from gasification of vineyard pruning at 900-950°C.	Growing media component for nursery grapevine production.	0, 10%, 20%, 40% vol. of biochar.	Greenhouse study in pots.	BC applied at 10% enhanced plant-growth response, particularly expressed at the shoot stage.	Ronga et al. (2019).
*Vitis vinifera* L. (cv. Grüner Veltliner).	Biochar from softwood chips and cereal husk.at 480°C.	Remediation of Cu-polluted soils.	1 to 6 kg m^-2^ of biochar (d.w.).	Greenhouse study in soil columns.	Biochar enhanced Cu mobilization in a neutral vineyard soil but reduced the ecotoxicologically relevant Cu^2+^ fraction.	Soja et al. (2018).
*Vitis vinifera* L. (cv. Chardonnay).	Biochar from poultry litter at 550°C.	Fungal-disease suppression and control of nematode population.	6.9 t ha^−1^ of biochar (d.w.).	Field trial (3 years),vineyard (5 years old).	Decreased plant-parasitic nematode population, andincreased free-living (non-plant-parasitic) nematode population.Possible role of biochar porous structure protecting bacteria and fungi from predators.	Rahman et al. (2014).
*Vitis vinifera* L. (Red Globe).	Bamboo biochar.	Microbial inoculum (*Pseudomonas putida*).	250 – 500 g biochar per tree (d.w.).12.5 g biochar mL^−1^ inoculum.	Field trial (1 year),vineyard (8 years old).	Inoculated biochar improved the yield and quality of the grapefruit.Enhanced soil properties and altered soil microbial community structure.	Wei et al. (2020).
*Vitis vinifera* L. (Red Globe).	Wood vinegar from a hardy rubber tree at 550°C.	Inhibition of grey mold infection (*Botrytis cinerea)* during grape storage.	Grapes soaked in wood vinegar diluted 600, 400, and 200 times for 15 s and left to air-dry.	Laboratory conditions.	Inhibitory effect on the disease *Botrytis cinerea* during grape storage by stimulation of antioxidant defense-related enzymes.	Chen et al. (2020).

d.w., dry weight.

Biochar may also act as a biostimulant when used as a carrier of inoculum for microorganisms (Hale et al., 2015; Głodowska et al., 2016; Hardy and Knight, 2021), as a suppressor of plant disease (Graber et al., 2010; Debode et al., 2020), and as a coating for novel biochar-fertilizer composites (Joseph et al., 2013) or slow-release fertilizers (An et al., 2020). However, these applications have not been fully explored in vineyards. Hale et al. (2015) demonstrated in a soil incubation experiment that biochar might support high population densities of the plant growth-promoting rhizobacteria (PGPR) *Enterobacter cloacae*. Interestingly, chemical properties of the biochar (particularly pH and N content) affected initial inoculum survival, but physical properties (surface area and porosity) were mainly associated with later survival after soil application (Hale et al., 2015). Biochar did not negatively affect rhizobacterium activity, which may occur due to bacterial signaling compounds or bacteria-derived plant growth hormones binding to the biochar surface. Depending on feedstock and pyrolysis conditions, certain biochars with large surface area or high pH can adsorb or hydrolyze signaling molecules, disrupting soil microbe cell-to-cell communication (Gao et al., 2016).

Similarly, Zhu et al. (2017) reported that biochar could modify communication between rhizosphere microbial communities and plant roots, affecting plant response to soil-borne pathogens (Harel et al., 2012; Akhter et al., 2015). Graber et al. (2010) proposed that induced resistance against pathogens observed in both tomato and pepper following biochar application may be due either to a shift in soil microbial populations following biochar addition, or to the release of chemicals from biochar toxic to pathogens. The utilization of beneficial microorganisms as biostimulants is discussed in greater detail below, and other mechanisms by which biochar can act as a disease-suppressing agent have been previously summarized by Bonanomi et al. (2015) and Graber et al. (2014).

Pyroligneous acid is often applied as a foliar spray or soil drench (Mungkunkamchao et al., 2013), or in combination with biochar (Zhang et al., 2020). Zhang et al. (2020) demonstrated the benefit of applying biochar and pyroligneous acid for increased blueberry yield and nutritional quality, by enhancing soil organic matter and nutrient availability. In terms of vineyard pest management, Chen et al. (2020) studied the ability of pyroligneous acid to inhibit grey mold (*Botrytis cinerea*) infection of table grape cultivar ‘Red Globe’. The application of diluted pyroligneous acids (200- and 400-fold dilution) improved grape resistance to grey mold, likely through stimulation of antioxidant defense-related enzyme activities, including those of superoxide dismutase, peroxidase, and ascorbate peroxidase. However, other investigations of pyroligneous acid use in vineyards are lacking.

The application of biostimulants from pyrogenic materials may help vineyards adapt to climate change, especially in a scenario of water shortage which may be particularly detrimental to viticulture (Maienza et al., 2017). Fascinatingly, the positive impacts of biochar on vineyard soil fertility are maintained in the long term (7 years), even after a single application (Giagnoni et al., 2019). This benefit has also been identified for improving the growth and symbiotic performance of other plants (lupin, *Lupinus angustifolius*) under drought stress conditions (Egamberdieva et al., 2017).

## Microorganisms as biostimulants

### Plant growth-promoting rhizobacteria

Certain prokaryotic soil microorganisms can establish beneficial relationships with plants. Coined ‘plant growth-promoting rhizobacteria’ (PGPR) (Kloepper and Schroth, 1979), these microbes can enhance plant growth and development through several direct and indirect processes, performed at different plant growth stages (Vorholt, 2012; Ruzzi and Aroca, 2015; Castellano-Hinojosa and Bedmar, 2017). According to Chauhan et al. (2015), the main PGPR traits are biofertilization and phytostimulation (through the excretion of phytohormones such as indole-3-acetic acid [IAA], cytokinins, and gibberellins), tolerance to biotic and abiotic stress (via 1-aminocyclopropane-1-carboxylate [ACC] deaminase activity), and biopesticide and biocontrol activity (production of antibiotics, lytic enzymes, hydrogen cyanide [HCN], and volatile organic compounds [VOCs], among others) (Chauhan et al., 2015; Kumar-Jha and Saraf, 2015; Goswami et al., 2016; Oleńska et al., 2020). Additionally, PGPRs can have synergistic interactions with the endophytic grape microbiome (Vandana et al., 2021).

Bacteria must first be isolated and cultured prior to their utilization as PGPRs. For use in viticulture, several authors have isolated viable PGPRs from the microbiome of *Vitis vinifera* L., which may then be re-applied as biostimulants in vineyards (Compant et al., 2011; Pinto and Gomes, 2016; Rezgui et al., 2016; Pacifico et al., 2019). The effectiveness of these PGPRs depends on several factors including environmental conditions, soil characteristics, and even crop variety (Pacifico et al., 2019). Nevertheless, PGPRs are a feasible tool for use in vineyards to effectively promote plant growth and protection (**Figure 1**).

Most studies of PGPRs in viticulture are focused on the initial stages of crop development in specific vine cultivars. Additionally, few studies testing strains at a field scale can be found (**Table 5**). Under *in vitro* and/or greenhouse conditions, Muganu et al. (2015); Köse et al. (2015), and Velásquez et al. (2020a) noted a wide variety of PGPR strains (*Ensifer, Burkholderia, Pseudomonas*, and *Bacillus*) were able to promote growth and root callusing percentage in four different *V. vinifera* cultivars: ‘Beyaz Çavus’, ‘Italia’, ‘Cabernet Sauvignon’, and ‘Chardonnay’. Köse et al. (2003) observed *Bacillus* strains BAI6 and OSU142 increased rooting in 41B (a hardy cross between old-word *V. vinifera* and new-world *V. berlandieri*) rootstocks. Similarly, Sabir (2013) and Sabir et al. (2017) applied *P. putida* BA-8 and B. simplex T7 to 41B hybrids and recorded a promotion of graft callusing, scion shoot growth, nursery survival rate, and fruitfulness. Salomon et al. (2016) tested a combination of strains *B. licheniformis* and *P. fluorescens* in order to improve the growth of *V. vinifera* cultivar ‘Malbec’ in greenhouse conditions. Also in a greenhouse, the cytokinin and auxin-producing *B. megaterium* M3 was applied in combination with *Agrobacterium rubi* A18 and *Alcaligenes eutrophus* Ca-639 to *V. vinifera*, grafted onto 41 rootstocks. Growth enhancement and increased grapevine pruning residue weight were noted (Salomon et al., 2017).

**Table 5:** An overview of the main effects of plant-growth-promoting rhizobacteria (PGPR) applied in viticulture.

Crop variety	PGPR strains	Properties	Application and dosage	Experimental condition	Effects	Reference
*Vitis vinifera* L. cv. Cabernet Sauvignon.	*Bacillus aryabhattai* JY17.	Phosphate solubilization.IAA.Siderophore.ACC deaminase.Chitinase.HCN (JY22).	Inoculation with 50 mL (10^8^ CFU mL^-1^) into the middle part of the seedling roots.	Greenhouse, transplanted after 3-4 leaves had grown on the seedlings, 28 L pots.	Increased plant height, stem thickness, root and shoot dry weight.	Liu et al. (2016).
	*Bacillus aryabhattai*JY22.					
*Vitis berlandieri* hybrids (41B and 1103P).	*Pseudomonas putida* BA-8.	Cytokinin synthesizer.	Scion node dipped into bacterial suspension (10^9^ CFU mL^-1^) for 1h.	Controlled greenhouse, and rootstocks.	Increased graft callusing, scion shoot growth, cane hardening, and nursery survival rate and fruitfulness	Sabir (2013).
	*Bacillus simplex* T7.	Auxin synthesizer.				
*Vitis vinifera* L. (Malvasia bianca lunga).	*Burkholderia* spp. strain IF25.	Biostimulation.Phosphate solubilization.Siderophores.IAA production.	Bi-nodal shoots dipped into 50 μL of bacterial inoculum.	*In vitro.*	Induced advanced rooting and high rooting percentage.	Muganu et al. (2015).
*Vitis vinifera* cv. Beyaz Çavus and Italia.	*Pseudomonas* BA8.	Auxin production.	Rootstocks dipped into bacterial suspension (10^9^ CFU mL^-1^).	*In vitro.*	Increased success and improvement of callusing rate, callusing degree, and full callusing rate in all rootstock-scion combinations.	Köse et al. (2015).
	*Bacillus* BA16.					
	*Bacillus* OSU142.					
*Vitis vinifera* cv. Cabernet Sauvignon	*Ensifer meliloti* TSA41.	Phosphate solubilization.Phytase.IAA production.	Plants inoculated at sowing with 10^6^ CFU g^-1^.	*In vitro.*	Enhancement of plant growth.	Velásquez et al. (2020a).
*Vitis rupestris* and 41B (*Vitis vinifera* x *Vitis berlandieri*).	*Bacillus* BAI6.	Plant-growth promotion.	Rootstocks dipped in bacterial solution (10^9^ CFU mL^-1^).	Mist chamber.	The two strains combined increased rooting in 41B.	Köse et al. (2003).
	*Bacillus* OSU142.					
*Vitis vinifera* cv. Malbec.	*Pseudomonas fluorescens.*	Enhancement of growth.Decrease of water loss rate by increasing ABA concentrations in leaves, inducing systemic responses.	Cuttings were submerged in bacterial suspension (10^6^ CFU mL^-1^).	Greenhouse.	Growth improvement and increased defense mechanisms to cope with biotic and abiotic stresses.	Salomon et al. (2016).
	*Bacillus licheniformis.*					
*Vitis berlandieri* hybrids (41B and 1103P).	*Bacillus licheniformis* Rt4M10.	Production of phytohormones.	Emerging roots of 15 days-old *in vitro* plants into bacterial culture (10^6^ CFU mL^-1^).	*In vitro.*	Increased shoot and root length, and leaf area. Induction of terpenes and ABA synthesis. Alleviation of drought and production of defense compounds.	Salomon et al. (2014).
	*Pseudomonas fluorescens* Rt6M10.			Greenhouse.		
*Vitis vinifera* L. cv. Alphonse Lavallée) plants grafted on 41 (*V. vinifera* cv. Chasselas ×*V. berlandieri*) rootstock.	*Bacillus megaterium* M3.	Auxin and cytokinin producing.N-fixing.Ca(HCO_3_)_2_ solubilizing.	Watering the plants with bacterial solutions (10^9^ CFU mL^-1^) one week after bud break.	Soilless growth system under controlled glasshouse conditions.	Enhanced shoot thickness and pruning residue weight of grapevines.	Sabir et al. (2017)
	*Agrobacterium rubi* A18.	Auxin and cytokinin producing.Ca(HCO_3_)_2_ solubilizing.				
	*Alcaligenes eutrophus* Ca-637.	Auxin producing.Ca(HCO_3_)_2_ solubilizing.				
*Vitis champini* (Ramsey).	Lactic acid bacteria:*Lactobacillus fermentum, L. Indolebutyric plantarum, L. Rhamnous, L. Delbrueckii.*	Plant-growth promotion.	EM•A (EM AGRITON) was applied to cuttings with different methods at different times and doses.	Greenhouse.	Increased rooting.	İşçı et al. (2019).
	Yeast: *Saccharomyces cerevisiae.*					
	Phototrophic bacteria:*Rhodopseudomonas palustrisBacillus subtilis.*					
*Vitis vinifera* L. cv. Chardonnay.	*Burkholderia phytofirmans* strain PsJN.	Endophyte.Enhancement of chilling resistance.	Nodal explants, immersed in the inoculum (10^6^ CFU mL^-1^).	Growth chamber.	Stimulation of growth and improvement of resistance to cold stress.	Barka et al. (2006).
*Vitis vinifera* L. cv. Malbec.	*Bacillus licheniformis.*	Plant-growth promotion.Protection against As(III).	Stem-base inoculation with 50 mL of bacterial suspensions (10^6^ CFU mL^-1^).	Greenhouse, two-year-old plant sprouts, 10 L pots, 50 mM NaAsO_2._	Stimulated growth and fruit yield, reducing AsIII toxicity indicators.	Funes-Pinter et al. (2018).
	*Micrococcus luteus*					
	*Pseudomonas fluorescens.*					
*Vitis vinifera* L. cv Victoria/*Vitis vinifera* L. cv Viorica.	*Agrobacterium radiobacter.*	Plant-growth promotion.Protection against Cu.	Bacterial suspensions were applied into the soil during the planting of rooted cuttings (10^7^CFU mL^-1^).	Growing platform.	Enhancement of the resistance of grape seedlings to Cu excess in soil.Decreased need for mineral fertilizer.	Veliksar et al. (2019).
	*Pseudomonas putida X.*					
	*Bacillus subtilis L.*		Foliar fertilization.			
*Vitis vinifer*a cv. Syrah*Vitis vinifer*a cv. Sauvignon.	*Paenibacillus illinoisensis.*	IAA production.ACC deaminase.Phosphate solubilization.Nitrogen fixation.	The bacterial suspension (10^8^ CFU mL^-1^) was applied by root soaking and irrigation.	Field experiment. One-year-old Syrah plantlets were grafted onto 1103P rootstock, one-year-old Sauvignon plantlets grafted on SO4 rootstock, and 17-year-old Syrah plants grafted onto Fercal rootstock.	Increased plant growth, number of grape brunches, and grape production.	Rolli et al. (2017).
	*Pseudomonas putida.*	IAA.ACC deaminase.P solubilization.Siderophores.Ammonia production.				
	*Bacillus subtilis.*	ACC deaminase.Exopolysaccharides.Siderophores.N fixation.Protease synthesis.				
	*Delfia tsuruhatensis.*	IAA.ACC deaminase.P solubilization.Exopolysaccharides.				
	*Pseudomonas fluorescens.*	IAA.ACC deaminase.P solubilization.Siderophores.N fixation.				
	*Pseudomonas rhodesiae.*	IAA.ACC deaminase.P solubilization.Siderophores.				
	*Achromobacter xylosoxidans.*	ACC deaminase.Siderophores.				
*Vitis vinifera* cv. Сodrinskii/*Vitis vinifera* cv. Presentable.	*Azotobacter chroococcum.*	Plant-growth promotion.	Inoculation of rooted cuttings in 11L Plastic pots (10^7^ CFU mL^-1^)	Growing platform.	Decreased need for mineral fertilizer and improvement of photosynthetic activity and plant growth.	Veliksar et al. (2017).
	*Bacillus subtilis.*		Spray with bacterial metabolites.	Vine nursery.		
	*Pseudomonas fluorescens.*					
Italian grape species grafted on 5BB rootstock (*Vitis berlandieri*×*V. riparia*).	*Bacillus megaterium* RC07.	Plant-growth promotion.	Inoculation of seedlings(10^8^ CFU mL^-1^).	Field trial.	Triple inoculation and single inoculation improved plant-growth parameters.	Erdogan et al. (2018).
	*Pseudomonas putida* RC06.					
	*Bacillus subtilis* RC11.					
	*Pseudomonas putida* FA19d.					
	*Pseudomonas fluorescens* RC77.					
	*Bacillus subtilis* RC63.					
	*Serratia marcescens* K2f.					
*Vitis vinifera* (Red Globe).	*Pseudomonas putida* Rs-198.	Plant-growth promotion.	Bacterial suspension (1.8 10^13^ CFU mL^-1^) at different doses. Application at dich around (close to 10 cm from each stem) old plants.	Field experiments. Old plants. One-season field experiment in the perennial vine.	Promoted alkaline phosphatase and invertase activity, increased the amount of available phosphorus and enhanced the growth and quality of the grape.	Lu et al. (2020).
Rootstock Kober 5BB clone ISV1 (*Vitis berlandieri* x *Vitis riparia*).	*Pseudomonas protegens* MP12.	IAA.Phosphate solubilization.ACC Deaminase.Ammonia production.	Bacterial suspension (10^8^ CFU mL^-1^).	*In vitro.*	Biocontrol agent and *in vitro* antimicrobial activity against several fungal pathogens.	Andreolli et al. (2016).
*Vitis vinifera* cv. Chardonnay.	*Burkholderia phytofirmans* PsJN.	Endophyte.Induction of plant resistance to biotic and abiotic stress.	Spray bacterial suspension (10^6^ CFU mL^-1^) on 4-week-old grapevine leaves of plantlets.	*In vitro*-plantlets, growth chamber.	Protection against *Botrytis cinerea.*	Miotto-Vilanova et al. (2016).
*Vitis vinifera* cv. Malbec.	*Microbacterium imperiale* Rz19M10.	Stimulation of the synthesis of terpenes.	0.25 mL of Bacterial inoculum (10^6^ CFU mL^-1^).	*In vitro.*	Reduction of lesion diameter provoked by *Botrytis cinerea*,systemic response induction, and increased terpene production.	Salomon et al. (2016).
	*Kocuria erythromyxa* Rt5M10.					
	*Terribacillus saccharophilus* Rt17M10.					
*Vitis vinifer*a cv. Chardonnay 7535.	*Pseudomonas fluorescens* CHA0.	Induction of systemic resistance2;4-diacetylphloroglucinol.Pyoluteorin.Pyrrolnitrin.AprA, exoprotease.HCN.	Dipping the root system in a 20 mL bacterial suspension (10^7^ CFU mL^-1^).	*In vitro*, four-week-old grapevine plantlets.	Induced resistance in grapevine against *Botrytis cinerea.*	Verhagen et al. (2010).
	*Pseudomonas aeruginosa* 7NSK2.	Induction of systemic resistance.Pyoverdin.Pyochelin.Pyocyanin.Salicylic acid.				
*Vitis vinifera* cv. Muscat d’Italie.	*Bacillus subtilis* B6.	Antibiotic production.	Bacterial suspension (10^8^ –10^9^ CFU mL^-1^) application in stem cutting.	*In vitro* and vineyard.	Protection against Grapevine Trunk Diseases (GTDs) and fungi antagonists.	Rezgui et al. (2016).
*Vitis vinifera* ‘Chardonnay’ (clone 7535).	*Pseudomonas* sp. Sn48.	Endophytic.Reduced gall formation by *Agrobacterium tumefaciens.*	Root dipping in bacterial suspensions (10^8^ CFU mL^-1^) for 1–2 min.	*In vitro* and growth chamber.	Induction of defenses against *Agrobacterium tumefaciens.*	Asghari et al. (2020).
	*Pantoea* sp. Sa14.					

In a rare field-scale experiment, Rolli et al. (2017) applied several bacteria from the genera *Paenibacillus, Pseudomona, Bacillus, Delftia*, and *Achromobacter*, to *V. vinifera* cultivars ‘Syrah’ and ‘Sauvignon’. Increased plant growth, number of grape brunches, and grape production were recorded. Veliksar et al. (2017) applied strains of *Azotobacter*, *Pseudomonas*, and *Bacillus* to two *V. vinifera cultivars* in Moldova, and observed improved photosynthetic activity, as well as reduced mineral fertilizer requirements. Erdogan et al. (2018) achieved similar improvements with combinations of a wide range of PGPR strains in grafted rootstocks of *V. berlandieri* and *V. riparia* in Turkey. In China, Lu et al. (2020) observed enhanced growth and final grape quality, following the application of *P. putida* Rs-198 to a perennial variety.

In addition to general growth and quality enhancement, inoculation with PGPRs may also induce grape tolerance against biotic and abiotic stresses. For example, the application of phosphate-solubilizing bacteria isolated from the *Vitis* rhizosphere resulted in improved plant development when grown in saline-alkaline soils (Liu et al., 2016). Salomon et al. (2014) observed both drought alleviation and production of defense compounds in the grape cultivar ‘Malbec’. Barka et al. (2006) found that *Burkholderia phytofirmans* PsJN could effectively protect Chardonnay grapes against cold stress.

Other studies focus on using PGPR strains to alleviate heavy metal stress. Funes-Pinter et al. (2018) identified *B. licheniformis, Micrococcus luteus*, and *P. fluorescens* as effective species for elevating growth and fruit yield in plants when grown in the presence of arsenic (As III). Veliksar et al. (2019) used strains of *Agrobacterium radiobacter, P. putida*, and *B. subtilis* to increase resistance against high soil concentrations of copper, and reduce the amount of mineral fertilizer needed.

Microorganisms may also be used as biocontrol agents against fungal pathogens in vineyard soils. For example, several studies have applied bacterial strains in order to protect vineyards from the common vine pest grey mold (*Botrytis cinerea*). Miotto-Vilanova et al. (2016) and Verhagen et al. (2010) found that several strains belonging to he *Pseudomonas*, *Burkholderia*, *Microbacterium*, *Kocuria*, and *Terribacillus* genera harbored plant growth and phytopathogen-control traits against *B. cinerea*. Andreolli et al. (2016) and Rezgui et al. (2016) also tested strains of *P. protegens* and *B. subtilis* against fungal pathogens, with the latter obtaining good results in field trial conditions. These studies show that a vast myriad of bacterial-borne traits can be effective for biocontrol, including the production of antibiotics, terpenes, pigments, and proteases, as well as inducing resistance against biotic stresses. As an indication of the high potential for PGPRs to combat plant biological stress, Asghari et al. (2020) applied *Pseudomonas* and *Pantoea* sp. and noted lowered gall formation produced by *Agrobacterium tumefaciens*.

As shown above, PGPRs have so far primarily been researched *in vitro*, and in greenhouse trials. Few studies exist at the field scale and any practical yield benefit from these microorganisms has yet to be quantified (Bashan et al., 2014). Other optimizations must be performed before any selected consortia can be developed into commercial products. The survivability and persistence of the applied strains must be quantified not only in the soil along with any competitive/negative effects from the native rhizosphere microbiome but also in stable storage conditions. European legislation restricting the application of chemical substances to crops has been passed relatively recently (Regulation EC 1107 (2009). It is therefore alarming that only a single reviewed study concerned the development of commercial bioinoculants; İşçı et al. (2009) applied consortium EM·A (comprised of lactic acid, phototropic bacteria, and yeasts; EM AGRITON Ltd., Belgium) to *V. champini*, and recorded improved rooting.

### Mycorrhizae

More than 400 MYA, the presence of mycorrhiza in soil was essential for the colonization of terrestrial environments by plants due to their ability to provide stress tolerance and soil resources *via* symbiosis (Heckman et al., 2001). This adaptation continues in the majority of plants on Earth, including grapevines (Trouvelot et al., 2015). Roots of grapevines form a particular type of mycorrhizal symbiosis called arbuscular mycorrhizae, characterized by the penetration and internal colonization of plant root cells by fungal hyphae (Trouvelot et al., 2015). Arbuscules form within the plant roots and serve as the exchange site for various metabolites (Trouvelot et al., 2015). Arbuscular mycorrhizal fungi (AMF) are widely known for being able to enhance the uptake of P in the host roots, a nutrient that is typically limiting in cropping systems like vineyards (Smith et al., 2011; Van Geel et al., 2017). However, they also provide increased plant uptake of N and other limiting elements including trace metals Fe, Mn, Cu, and Zn (Clark and Zeto, 2000), which are also critical to plant health (**Figure 1**). This is accomplished by greatly extending the root system’s exploration and exploitation area (Smith and Read, 2008; Trouvelot et al., 2015), simultaneously increasing plant access to water sources that would be otherwise inaccessible (Al-Karaki and Clark, 1998). This feature may be particularly advantageous in rain-fed vineyards growing in nutrient-poor soils, which are widespread in most traditionally wine-producing regions such as the Mediterranean, Middle East, and Caucasian regions (**Table 6**).

**Table 6:** An overview of the main effects of mycorrhizal inoculants applied in viticulture.

Crop variety	Applied material and properties	Dosage, the form of application, and stage of plant development	Experimental condition	Effects	Reference
*Vitis vinifera* cv. Pinot noir.	Mixed inoculum of 3 AMF isolated from a vineyard and recultured with *Sorghum bicolor*: *Scutellospora calospora* INVAM# OR219, *G. mosseae* INVAM# OR218, and *Glomus* sp. INVAM#215.	A mixture of soil with AM fungal spores, hyphae, and colonized root fragments. 20 g of inoculum plant^-1^.	Potted plants in a greenhouse.	Vine growth was dependent on AMF in one soil, but inoculated and non-inoculated vines grew equally well in another soil. Increase in plant dry mass with AMF due to enhanced P uptake (833% increase). The uptake of most other nutrients was also enhanced by AMF in the first soil.	Schreiner (2007).
*Vitis vinifera* cv. Pusa Navrang.	Six single strains and a mixture of AMF (*G. manihotis*, *Glomus mosseae*, and *G. gigantean*) recultured with *Chloris guyana*: *Acaulospora laevis*, *A. scrobiculata*, *Entrophospora colombiana*, *Gigaspora gigantea*, *Glomus manihotis*, and *Scutellospora heterogama*.	A mixture of soil with AM fungal spores, hyphae, and colonized root fragments. 20 g of inoculum plant^-1^.	Micropropagated plantlets in a greenhouse.	Enhanced survival and improved tolerance against stresses. Improved physiological and nutritional status and higher relative water content and photosynthetic rate. Higher concentrations of N, P, Mg, and Fe. Better hardening.	Krishna et al. (2005).
*Vitis vinifera* cv. Tempranillo.	GLOMYGEL Vid, Olivo, Frutales (Mycovitro S.L., Pinos Puente, Spain): Culture of AMF *Rhizophagus intraradices.*	8 mL of diluted mycorrhizal inoculum plant^-1^ (equivalent to 2000 propagules).	Cuttings are planted in 6.5-L plastic pots.	AMF inoculation improved parameters linked to phenolic maturity such as anthocyanin content and increased antioxidant activity under elevated temperature.	Torres et al. (2016).
*Vitis vinifera* cv. Sangiovese.	*Funneliformis mosseae* IMA1.	A mixture of soil with AM fungal spores, hyphae, and colonized root fragments and autoclaved peat in a proportion of 1:4 v/v + 2 mL of *F. mosseae* IMA1 inoculum filtrate.	Explants were cultivated in 150 mL sanitized pots.	Greater emission of volatiles related to plant defense and water stress.	Velásquez et al. (2020b).
*Vitis vinifera* cv. Viosinho.	*Funneliformis mosseae* inoculum (isolate BEG95, Symbiom^®^, Czech Republic).	10 g of inoculum plant^-1^ buried in ditches and mixed with soil and rye seeds.	Field experiment.	Greater establishment of new mycorrhizal taxa in vine roots. Greater photosynthetic efficiency after a heat wave. Compensation for water competition with cover crops.	Nogales et al. (2021).
*Vitis vinifera* cv. Cabernet Sauvignon.	Commercial inoculum Mykoflor (Mykoflor, Polland).	20 mL of suspension under the vine roots (~2000 propagules).	Field experiment.	Improved leaf gas exchange.Higher yield and number of clusters. Greater polyphenols and anthocyanins.	Karoglan et al. (2021).

In addition to their role in plant water and nutrient uptake, AMF contributes to the biosynthesis of a wide range of molecules such as vitamins and hormones needed to support the metabolism and health of plants (Strzelczyk et al., 1991), although this is poorly investigated in the context of vineyards. Arbuscular mycorrhizal fungi can also protect grapevines against harmful pathogens and pests, including the root-knot forming nematode *Meloidogyne incognita*, through the induction of a defense response involving enzymes like chitinases (Li et al., 2006). Moreover, AMF contributes to the development of a healthy rhizosphere community (i.e., a microbially diverse community that is functionally linked to the plant), which in turn may confer tolerance/resistance against a range of biotic and abiotic stresses (reviewed above), including heat, drought, salinity, pathogenic infection, and pests (Fitter and Garbaye, 1994; Gryndler, 2000; Jeffries et al., 2003; Gupta et al., 2018). Given their importance in vine nutrition and pathogen response, AMF are a critical pillar of healthy, functioning vineyards (Trouvelot et al., 2015), especially in the current context of widespread environmental degradation and climate change. In addition to direct benefits to plant health, mycorrhizae may also provide important ecosystem services, including increasing potential carbon sequestration of vineyard soils and reducing erosion (Trouvelot et al., 2015).

Despite these benefits, mycorrhizal symbiosis is frequently disrupted in croplands due to intensive management, including excessive tilling, which breaks the orderly structure of soil aggregates and fungal networks (Gosling et al., 2006; Bowles et al., 2017; Porter and Sachs, 2020), although this has been poorly investigated in vineyards (Winter et al., 2018). Biocides can also negatively affect mycorrhizal fungi, with deleterious consequences on the establishment of symbiosis (Gosling et al., 2006; Zaller et al., 2018), while synthetic fertilizers disrupt the mycorrhizal association due to the ablation of nutritional constraints, such as N and P (Gosling et al., 2006; Van Geel et al., 2017). Regenerating proper vine-mycorrhizal balance and function in degraded vineyards is thus a priority, which may yield many benefits to both growers and the wider society.

Regeneration of the vine-AMF interaction can take place through various means, which should be considered holistically in terms of impact on total vineyard management. One such strategy is the inoculation of grapevines with one or more strains of mycorrhizal fungi previously selected for their ability to colonize vine roots (Linderman and Davis, 2001; Trouvelot et al., 2015). One aspect in which inoculation with mycorrhizal biostimulants might be particularly important is to minimize the growing threat of trunk diseases (Petit and Gubler, 2006; Holland et al., 2019), which may be linked to widespread alteration of plant-microbial associations. It has been recently suggested that declines observed in woody plants are related to microbiome modifications or imbalances (i.e., dysbiosis) (Porter and Sachs, 2020). Arbuscular mycorrhizal fungi known to help re-establish symbiosis include various strains of *Glomus intraradices*, such as *G. intraradices* BEG72 (Nogales et al., 2008) and INVAM CA501 (Petit and Gubler, 2006).

Proper timing may be critical when inoculating plants with mycorrhizal biostimulants (Sohn et al., 2003), but there is little information for vineyards. It is suggested that rootstocks be submerged in an AMF spore solution before planting, but variable success is reported. For example, a study was carried out on *Vitis rupestris* cv. St. George using *G. intraradices* and reported that pre-inoculated plants were less susceptible to black foot disease (Petit and Gubler, 2006), while (Holland et al., 2019) observed no difference between inoculated and uninoculated plants. Some studies have also reported clear effects in the greenhouse, but not under realistic field conditions (Rosa et al., 2020). Other ways to add AMF to vineyards include the direct spraying of commercial biofertilizers on adult plants, through the addition of granules containing spores, and potentially also the translocation of whole soil inoculants from previously selected locations particularly diverse in terms of AMF such as forests. This latter approach is underexplored in vineyards. Perhaps contributing to variability in inoculation success is the difficulty in altering the pre-established microbiome of adult plants. Moreover, the mixed positive and neutral effects of inoculating vines with AMF, together with the fact that some (but not all) mycorrhizal fungi show a certain degree of host specificity (Campos et al., 2018), suggest the importance of screening AMF strains against potential compatible host vine cultivars/rootstocks prior to inoculum development (Schreiner, 2007). Finally, the vine-AMF symbiosis is context dependent and may be linked to factors critical for productive vineyards including soil fertility and other properties (e.g., soil organic matter content, pH, and texture) (Trouvelot et al., 2015). For example, the presence of a well-developed community of cover crops in vineyard inter-rows may favor inoculated AMF establishment, by providing an additional host crop and continuous reservoir for supplying adjacent grapevines (Winter et al., 2018).

## Biological control agents

Climate change simulations predict that the fitness of crop insect pests will increase beyond 30° latitude North and South of the equator (Santos et al., 2020). Indeed, a three-decade study observed shifts in the phenology of grape berry moths (*Eupoecilia ambiguella*, *E. viteana*, and *Lobesia botrana*), distribution ranges of leafhoppers (*Scaphoideus titanus* Ball, a common vector of grapevine diseases), and range expansion of grapevine mealybugs (*Planococcus ficus*) (Reineke and Thiéry, 2016).

The effective control of crop pests is a continuous process. For native and newly introduced pests, the adoption of novel biological control agents (BCAs) is needed for sustainable alternative pest management (**Figure 1**). BCAs are living organisms antagonistic against pests. By definition, biological control must involve: 1) a biocontrol agent, 2) a pest to be controlled, and 3) a farmer or a stakeholder benefitting from the pest control (Stenberg et al., 2021). Organisms such as insects or mites, bacteria, fungi, and nematodes are used to control weeds or pests and diseases of cultivated plants (Ehlers, 2011). Viruses are not living entities, but contain structural biological components such as nucleic acids and proteins and are therefore also considered BCAs (Stenberg et al., 2021). Semiochemicals (chemical molecules produced by organisms that modify the behavior of other living beings, i.e., bio-communication) and plant extracts that can act directly on a pathogen or pest are other options for BCAs (Ehlers, 2011). Biological control is an interaction between at least two organisms, and success is therefore influenced by many factors including climate, reproduction mode and rate, food availability, and others. Holistically considering the total impact of all dynamic factors together when designing a balanced pest management plan is referred to as integrated pest management (IPM). BCAs are a main component of IPM, and together, are becoming increasingly utilized in agriculture.

Biocontrol agents may be naturally present in the agroecosystem (e.g. a native population of soil microbes antagonistic to plant-parasitic nematodes), or first grown *in vitro* and then released (Sharma et al., 2009; Stenberg et al., 2021). Grapevine is a perennial crop and harbors a large microbiome in both the rhizosphere and stem tissues. Additional microorganisms which act to balance the microbiota are also found in the phyllosphere and fructosphere (Ranade et al., 2021). Many of these microorganisms act as BCAs and are a promising ecological strategy for disease control (Carro-Huerga et al., 2021).

The cropping system (e.g. conventional vs. organic farming) also influences the potential number of biocontrol agents which may be isolated for application. Cordero-Bueso et al. (2017) evaluated the biocontrol potential of 230 grape yeast isolates from different cultivation systems. The fractions with the most candidates were isolated from wild vines (62.7%) and biodynamic vineyards (17.7%). The least number of candidates were isolated from organic (6.2%) and conventional systems (7.2%). Wild vine species may therefore serve as a valuable resource for bioprospecting future BCAs. Additional potential sources of candidates include other plants, insects, animal intestinal tracts, soils, and marine and freshwater environments (Kurtzman et al., 2011).

BCAs can have several mechanisms of action against pathogens. During resource limitation, BCAs may outcompete detrimental microbes in terms of space (niches), nutrients, water, and/or light (Wang et al., 2018; Almeida et al., 2020). Other mechanisms include iron depletion (Sipiczki, 2006; Cordero-Bueso et al., 2017); production of lytic enzymes (Cordero-Bueso et al., 2017; Cabañas et al., 2020); production of volatile organic and semio-compounds antagonistic against pests (Cabañas et al., 2020; Don et al., 2021); resistance induction of the host plant (Arras, 1996; Jeandet et al., 2002; Maachia et al., 2015; Haidar et al., 2016); direct parasitism; tolerance of reactive oxygen species (ROS) (Aziz et al., 2003; Jamalizadeh et al., 2011); biofilm formation (Cabañas et al., 2020); synthesis of pathogenesis-related proteins (Chan and Tian, 2006); and antibiotic production (Maachia et al., 2015; Cordero-Bueso et al., 2017).

Production of extracellular mucilage produced by microbial antagonists throughout host cells may be linked to cell adhesion, and contain biochemical elicitors to signal defense responses (El-Ghaouth et al., 1998). Fragments of yeast cell wall oligosaccharides have also been noted to possess elicitor potential.

In viticulture, most BCAs are applied to control fungal diseases and pests associated with insect and mite vectors. However, other biological controls have also been researched and suggested for other pests. For example, harmful mollusks (snails) in vineyards in cool and wet climates may be effectively controlled by the nematode *Phasmarhabditis hermaphrodite* (Schneider), as there are restrictions on the use of synthetic molluscicides (Egleton et al., 2021). BCAs are also being developed for the control of weed species prevalent in vineyards (Samad et al., 2017).

In a grapevine host, antibiosis and resistance induction were observed, attributed to the antagonism of *Bacillus subtilis* B29 against *Uncinula necator* (powdery mildew) and *Botrytis cinerea* (grey mold) (Maachia et al., 2015). An n-hexane extraction of the cell-free supernatant of *B. subtilis* B29 revealed the presence of 17 fractions through HPLC. Two fractions were considered antibiotics against *M. ramannianus* and *M. luteus*, based on their antimicrobial activity (Sihem et al., 2011). *B. subtilis* B29 and B27 have also been described as inducing host resistance through the high production of phenolic compounds, with a significant increase in hydroxycinnamic acid (Maachia et al., 2015).

Antagonism against pests alone does not necessarily make an organism a BCA. The applied fungi, yeasts, and bacteria must possess other characteristics to allow for practical use in the field. For example, a BCA with greater adaptability than the pest may allow for widespread niche colonization and higher competitive potential (El-Ghaouth et al., 2004). Additionally, prior to application, the BCA candidate should be extensively screened for the production of metabolites harmful to non-pest organisms, especially humans. In the specific case of biological control of molds on grapes following harvest, the BCA should not leave residue on the berries. After identifying the mechanism of antagonism, and confirming BCA adaptability and safety, appropriate tests following the guidelines of national regulatory bodies must be conducted before registration as a commercial inoculant.

The use of BCAs in agriculture began in the second half of the 20th century, and several candidates have been identified. Although many researchers have since evaluated their pest control potential, few inoculants reach commercialization. *Bacillus subtilis* is one of the most widely applied fungicidal BCAs (Garrido and Botton, 2020). During the 2020/2021 harvest in Brazil, 182 commercial brands of synthetic fungicides were available for application to vineyards, compared to 28 biological fungicides (**Table 7**). Only two genera were represented, *Bacillus* and *Paecilomyces*, with a predominance of *Bacillus* (mostly *B. subtilis*). Other species of *Bacillus* were *B. amyloliquefaciens*; *B. licheniformis*; *B. methylotrophicus*; and *B. pumilus*. *Paecilomyces lilacinus* has been cited as an active ingredient in seven commercial brands. Most are composed of a single species and strain.

**Table 7:** Principal biological control agents (BCAs) applied in viticulture in Brazil.

Trade (and company) names	BCA	Concentration	Dosage	Target pathogens	Application
NO-NEMA^®^ (Biovalens Biotecnologia)	*Bacillus amyloliquefaciens*	42 (3 x10^9^ UFC mL^-1^)	0.5 - 4.0 L ha^-1^	Nematodes (several species)	Soil/Seed
DURÁVEL^®^ (BASF)	*Bacillus amyloliquefaciens MBI600*	110 (5.5 x 10^10^UFC g^-1^)	0.5 - 1.0 kg ha^-1^	*Botrytis cinerea*	Soil/Aerial
ECO-SHOT^®^ (IHARA)	*Bacillus amyloliquefaciens* cepa D-747	250 (5 x 10^10^ UFC g^-1^)	1.0 - 4.0 kg ha^-1^	*Sclerotinia sclerotiorum, Colletotrichum gloeosporioides*	Foliar/fruit immersion
NEMA III	*Bacillus amyloliquefaciens*	42 (3 x10^9^ UFC mL^-1^)	0.5 - 4.0 L ha^-1^	Nematodes (several species)	Soil
NEMACONTROL^®^ (Simbiose^®^)	*Bacillus amyloliquefaciens*	30 (5 x 10^9^ UFC mL^-1^)	0.5 - 1.0 L ha^-1^	*Pratylenchus brachyurus*	Seed
PFC-CONTROL	*Bacillus amyloliquefaciens*	30 (5 x 10^9^ UFC mL^-1^)	0.5 - 1.0 L ha^-1^	*Pratylenchus brachyurus*	Seed
QUARTZ SC	*Bacillus amyloliquefaciens*	1,5 (3 x 10^9^ UFC mL^-1^)	1.0 - 2.0 L ha^-1^	*Botrytis cinerea*	Soil
PROFIX^®^ (Bula)	*Bacillus licheniformis, B. subtilis, Paecilomyces lilacinus*	200 + 200 + 200 (10^10^ UFC g^-1^)	50 - 70 g ha^-1^	*Meloidogyne incognita, Pratylenchus brachyurus*	Soil/Seed
ONIX^®^ (Lallemand)	*Bacillus methylotrophicus*	15 (10^9^ UFC mL^-1^)	6.0 L ha^-1^	*Meloidogyne javanica, Pratylenchus brachyurus*	Soil/Seed
ONIXog^®^ (Lallemand)	*Bacillus methylotrophicus*	15 (10^9^ UFC mL^-1^)	6.0 L ha^-1^	*Meloidogyne javanica, Pratylenchus brachyurus*	Soil/Seed
SONATA^®^ (Bayer CropScience)	*Bacillus pumilus* QST 2808	14.35 (10^9^ UFC g^-1^)	2.0 - 4.0 L ha^-1^	*Uncinula necator, Botrytis cinerea*	Aerial
BIO-IMUNE^®^ (Vittia Grupo)	*Bacillus subtilis* BV02	42 (3 x10^9^ UFC mL^-1^)	2.0 - 8.0 L ha^-1^	*Uncinula necator*	Foliar
BIOBACI/BIOBACI III^®^ (Biovalens Biotecnologia)	*Bacillus subtilis* BV09	7 (10^8^ UFC g^-1^)	1.5 - 6.0 L ha^-1^	*Meloidogyne* spp. *Fusarium oxysporum*	Soil
PRESENCE	*Bacillus subtilis* FMCH002*, Bacillus licheniformis* FMCH001	200 + 200 (10^11^ UFC g^-1^)	100 - 150 g 100 kg^-1^	*Meloidogyne javanica, Pratylenchus brachyurus*	Seed
SERENADE^®^ (Bayer CropScience)	*Bacillus subtilis* QST 713	13.68 (10^9^ UFC g^-1^)	2.0 - 4.0 L ha^-1^	*Botrytis cinerea, Colletotrichum gloeosporioides*	Soil/Aerial
RIZOS OG^®^ (Lallemand Soluções Agrobiológicas Ltda)	*Bacillus subtilis* UFPDA 764	3 (3x10^9^ UFC mL^-1^)	4.0 - 8.0 L ha^-1^	*Meloidogyne javanica, Pratylenchus brachyurus*	Seed
BIOBAC^®^ (Vital Brasil Chemical Indústria e Comércio de Produtos Químicos Ltda – ME)	*Bacillus subtilis* Y1336	500 (10^9^ UFC g^-1^)	0.8 - 1.0 kg 100 L^-1^	*Botrytis cinerea*	Aerial
QUATZO^®^ (FMC Química do Brasil Ltda)	*Bacillus subtilis, Bacillus licheniformis*	200 + 200 (10^11^UFC g^-1^)	130 - 300 g ha^-1^	Nematodes (several species)	Soil
BN 40.001/19 (Ballagro)	*Paecilomyces lilacinus*	300 (7.5 x 10^9^UFC g^-1^)	1.92 kg ha^-1^	*Meloidogyne incognita*	Soil
MNG-02/14 (Agrobiológica Sustentabilidade)	*Paecilomyces lilacinus*	7 (10^5^ UFC g^-1^)	1.0 - 4.0 kg ha^-1^	*Meloidogyne incognita*	Soil
NEMAKILL^®^ (Grupo Clínica Agrícola)	*Paecilomyces lilacinus*	7 (10^5^ UFC g^-1^)	1.0 - 4.0 kg ha^-1^	*Meloidogyne incognita*	Soil
NEMAT^®^ (Agroindustrial Limsa)	*Paecilomyces lilacinus*	300 (7.5 x 10^9^ UFC g^-1^)	0.1 - 0.25 kg ha^-1^	*Meloidogyne incognita, Pratylenchus brachyurus*	Soil/Seed
NETTUS^®^ (Ballagro Agro Tecnologia Ltda)	*Paecilomyces lilacinus*	300 (7.5 x 10^9^ UFC g^-1^)	1.28 - 1.92 kg ha^-1^	*Meloidogyne incognita*	Soil
Purpureonyd Fr25 (TZ Biotec)	*Paecilomyces lilacinus*	200(6.5 x 10^7^ UFC g^-1^)	1 kg 15 ha^-1^	* Meloidogyne incognita*	Soil/Aerial
VINTEC (Belchim Crop Production)	*Trichoderma atroviride*	1 x 10^13^ UFC g^-1^	200 g. ha^-1^	Esca, *Phaeomoniella chlamydospora*, *Togninia minima* (*Phaeoacremonium aleophilum*), *Eutypa lata, Botrytis fuckeliana, B. cinerea Fomitiporia mediterranea, Podosphaera xanthii, Armillaria mellea*, and *A. gallica*	Aerial (pulverization) or scion wood immersion

Esca is caused by the association of the fungi *Phaeoacremonium minimum* and *Phaeomoniella chlamydospore* and is one of the main Grapevine trunk diseases (GTDs). Control of Esca is achieved mainly through BCAs, in which common biological agents are *Trichoderma* fungi (Chervin et al., 2022). *Trichoderma* atroviride-based products (CS1 by Vintec^®^ - Belchim Crop Production and I-1237 by Esquive^®^ - Agrauxine by Lesaffre) are often effective because they have multiple mechanisms of action: substrate competition, antibiosis, and mycoparasitism (Pertot et al., 2015; Belchim, 2022). For the control of powdery mildew (*Uncinula necator*), AQ10^®^ (*Ampelomyces quisqualis*) is used commercially (Benuzzi and Baldoni, 2000). Another biologically-controlled disease is downy mildew (*Plasmopara viticola*). *Trichoderma harzianum* (known as Trichodex^®^), acts against the oomycete by increasing lignin, callose, and hydrogen peroxide, in addition to upregulating the defense enzymes phenylalanine ammonia-lyase, peroxidase, and 1,3-glucanase (Kamble et al., 2021).

The choice of BCA is of paramount importance. Simultaneous with disease control, some BCAs can influence grape productivity and quality with variable effects on acidity, soluble solids content, and berry size. Malviya et al. (2022) cite significant differences in the acidity of vine fruits treated with three BCAs ranging from 3.7 to 4.2 and 4° Brix in the SST. Production per plant doubled when treated with BCAs and almost tripled when combined with BCA + sulfur.

As for insecticides and acaricides, 55 synthetic products and 38 biological commercial products have been authorized for grapevines. Twenty-six contain *Beauveria bassiana* as an active ingredient and three with *B. thuringiensis*. For the control of insects and mites, recommendations for predatory insects (one *Orius insidiosus* and one sterile male pupil of *Ceratitis capitata*), predatory mites (four *Neoselulus californicus* and two *Phytoselulus macropilis* with AcMNPV virus), and one viral compound (AcMNPV virus, ChinNPV Virus, HearNPV Virus, SfMNPV Virus) are available.

Many antagonists with BCA potential are studied with increasingly efficient and cost-effective techniques. In addition to bioprospecting for biopesticides, valuable antibiotics may also be obtained from these organisms. Decreased BCA production costs and improved management techniques that increase and prolong BCA effects will allow for scaling their use in viticulture. Thus, biological products with multiple benefits (controlling disease(s), promoting plant growth, and increasing grape quality) are compliant with the sustainable development objectives of the 2030 agenda of the United Nations (UN). The reduction of synthetic chemical inputs in this manner can simultaneously reduce contamination throughout the production chain and ecosystem.

Future studies should investigate other applications of BCAs, such as the use of parasitic nematodes (*Phasmarhabditis hermaphrodita*) or earthworms (*Lumbricidae*) for pest control against snails and slugs in vineyards.

## Conclusions and future prospects for investigation

The application of biostimulants and BCAs may allow for improved sustainable viticulture and may serve as alternatives for chemically synthesized agronomic inputs, thereby reducing the negative environmental impact of pesticides and fungicides. The positive effects on plant growth are summarized in **Figure 1** and vary widely depending on the type of biostimulant applied to the crop. The optimization of these materials is necessary for a successful application, by considering biostimulant concentration and dosage effects, plant developmental stage, climatic/environmental conditions, and experimental setup.

Therefore, the main limitations and areas for further investigation for future biostimulant and BCA optimization in sustainable viticulture are listed below:

- A) Manufacturing and commercialization
- Harmonization of legislation and lack of regulations on product quality
- Competitive commercialization costs
- Availability of raw materials used for their manufacturing, especially the lack of quality material (i.e. heterogeneous composition)
- Storage and effectivity duration (especially for biological products)
- B) Application
- Lack of information at field-scale, and very few reports on the application in uncontrolled realistic conditions with positive results
- Effect variability on the unknown plant microbiome
- Effect variability on plant growth, depending on product dosage (concentration and number of applications)
- Effect variability depending on the mode of product application (foliar spraying, or *via* soil irrigation/fertigation)
- Effect variability depending on the stage of crop development
- Speculation remains as to underlying mechanisms associated with the biostimulant
- Comparative studies with agrochemicals as control
- Synergetic effects with other products and potential negative effects
- Lack of standard protocols depending on grape cultivar
- C) Environmental and practical issues
- Soil management
- Environmental and practical issues
- Soil management for effect optimization
- Integration with agronomical management (e.g. avoiding tillage for AMF)
- Short and long-term environmental impact (e.g. contamination of soil and watersheds)

## Author contributions

KJ: Conceptualization, Methodology, Writing - Original Draft; TLG: Writing - Review and Editing; PP-T, MS-M, YA, AD-A, AW, MG, MS, CP, JB, RO-H, MN, JR, and RA: Writing - Original Draft; SM and MD: Funding acquisition; GT: Writing - Original Draft, Writing - Review and Editing, Supervision and Funding acquisition. All authors approved the manuscript.

## Funding

KJ wishes to acknowledge financial support (3710473400); MS-M thanks to RTI2018-099417-B-I00 (Spanish Ministry of Science, Innovation and Universities cofunded with EU FEDER funds); JB wish to acknowledge the Conselho Nacional de Desenvolvimento Científico e Tecnológico/Brasil (CNPQ process number 309477/2021-2); RO-H is supported by the Ramón y Cajal program from the MICINN (RYC-2017 22032), PAIDI 2020 (Ref. 20_00323), AEI GGOO 2020 (GOPC-CA-20-0001), “José Castillejo” program from the “Ministerio de Universidades” (CAS21/00125) and PID2019-106004RA-I00/AEI/10.13039/501100011033. SM and GT thanks to Ministerio de Ciencia e Innovación (grant PID2020-114330GB-100). PAIDI2020 from Junta de Andalucía, grant P18-RT-1401 to SM, MD, and GT is also acknowledged. GT acknowledge the support of the publication fee by the CSIC Open Access Publication Support Initiative through its Unit of Information Resources for Research (URICI).

## Conflict of interest 

Author YA was employed by Chitose Laboratory Corp.

The remaining authors declare that the research was conducted in the absence of any commercial or financial relationships that could be construed as a potential conflict of interest.

## Publisher’s note

All claims expressed in this article are solely those of the authors and do not necessarily represent those of their affiliated organizations, or those of the publisher, the editors and the reviewers. Any product that may be evaluated in this article, or claim that may be made by its manufacturer, is not guaranteed or endorsed by the publisher.
